# Probing Methyl Group Tunneling in [(CH_3_)_2_NH_2_][Zn(HCOO)_3_] Hybrid Perovskite Using Co^2+^ EPR

**DOI:** 10.3390/molecules28030979

**Published:** 2023-01-18

**Authors:** Gediminas Usevičius, Andrea Eggeling, Ignas Pocius, Vidmantas Kalendra, Daniel Klose, Mirosław Mączka, Andreas Pöppl, Jūras Banys, Gunnar Jeschke, Mantas Šimėnas

**Affiliations:** 1 Faculty of Physics, Vilnius University, Sauletekio 3, 10257 Vilnius, Lithuania; 2 Department of Physical Chemistry, ETH-Zürich, Vladimir-Prelog-Weg 2, 8093 Zürich, Switzerland; 3 Institute of Low Temperature and Structure Research, Polish Academy of Sciences, Okólna 2, 50-422 Wroclaw, Poland; 4 Felix Bloch Institute for Solid State Physics, Leipzig University, 04103 Leipzig, Germany

**Keywords:** methyl group tunneling, hybrid perovskite, EPR, ESEEM

## Abstract

At low temperature, methyl groups act as hindered quantum rotors exhibiting rotational quantum tunneling, which is highly sensitive to a local methyl group environment. Recently, we observed this effect using pulsed electron paramagnetic resonance (EPR) in two dimethylammonium-containing hybrid perovskites doped with paramagnetic Mn^2+^ ions. Here, we investigate the feasibility of using an alternative fast-relaxing Co^2+^ paramagnetic center to study the methyl group tunneling, and, as a model compound, we use dimethylammonium zinc formate [(CH_3_)_2_NH_2_][Zn(HCOO)_3_] hybrid perovskite. Our multifrequency (X-, Q- and W-band) EPR experiments reveal a high-spin state of the incorporated Co^2+^ center, which exhibits fast spin-lattice relaxation and electron spin decoherence. Our pulsed EPR experiments reveal magnetic field independent electron spin echo envelope modulation (ESEEM) signals, which are assigned to the methyl group tunneling. We use density operator simulations to extract the tunnel frequency of 1.84 MHz from the experimental data, which is then used to calculate the rotational barrier of the methyl groups. We compare our results with the previously reported Mn^2+^ case showing that our approach can detect very small changes in the local methyl group environment in hybrid perovskites and related materials.

## 1. Introduction

**Figure 1:**
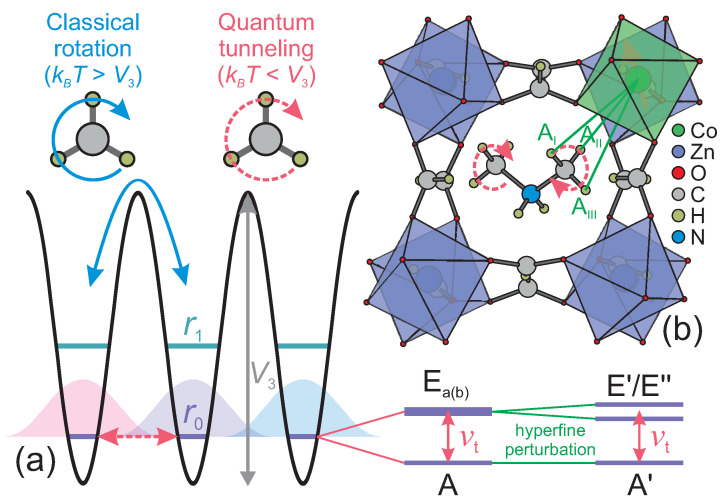
(**a**) Schematic energy level diagram of a methyl group rotor with rotational barrier $V_{3}$. At temperatures much lower than $V_{3}/k_{B}$, the group exhibits quantum tunneling enabled by the wavefunction overlap, which also splits the ro-librational levels into the A and $E_{a(b)}$ states. The splitting of the lowest level $r_{0}$ is called the tunnel splitting $\nu_{t}$. In the presence of an unpaired electron spin, the states are further perturbed due to the hyperfine interaction. (**b**) Low-temperature structure of DMAZn:Co. The hyperfine interactions between the paramagnetic Co^2+^ ion and protons of the nearest methyl group are indicated by green lines. Structural data taken from Ref. [7].

The methyl group is the most widely spread functional group found in various naturally occurring and synthetic compounds. At temperatures higher than its rotational barrier, this group exhibits classical reorientation dynamics around its three-fold symmetry axis, while at lower temperatures, it acts as a hindered quantum rotor for which only the ro-librational ground state is significantly populated [1,2,3,4]. In this case, stochastic rotation is replaced by rotational quantum tunneling caused by the wavefunction overlap of the three localized states [1,2] (see Figure 1a). The overlap also causes a splitting of the ro-librational ground state into the symmetric ground (A) and two antisymmetric degenerate excited ($E_{a(b)}$) states. For rotational barriers $V_{3}$ expected for aliphatic methyl groups, the tunnel frequency $\nu_{t}$ that splits these energy states depends approximately exponentially on $V_{3}$ and thus it can vary in a broad frequency range (GHz-kHz) making it a very sensitive probe to study the local methyl group environment such as chemical bonding, steric effects and defect-induced deformations [4,5,6].

The tunnel frequency can be obtained using different experimental techniques such as neutron scattering [4], heat capacity measurements [8], vibrational spectroscopy [9], NMR [1,3], continuous wave electron paramagnetic resonance (CW EPR) [10,11] and electron nuclear double resonance (ENDOR) [12,13,14,15]. The latter two techniques require paramagnetic centers to be naturally present or artificially introduced close to the methyl groups in the studied compounds. Recently, we demonstrated that pulsed EPR electron spin echo envelope modulation (ESEEM) experiments can be used to excite and detect the coherence between the tunnel-split states of the methyl groups providing a way to precisely determine $\nu_{t}$ in the MHz frequency range [16,17]. In contrast to ordinary ESEEM used to measure small hyperfine interactions with nearby nuclei [18], a distinct signature of the tunneling ESEEM is its independence of the magnetic field.

Our previous measurements of tunneling ESEEM were performed on [(CH_3_)_2_NH_2_] [Zn(HCOO)_3_] (DMAZn:Mn) and [(CH_3_)_2_NH_2_][Cd(N_3_)_3_] (DMACd:Mn) hybrid perovskites containing dimethylammonium (DMA) cations [17]. In these compounds, a tiny amount of diamagnetic metals (Zn^2+^ and Cd^2+^) was replaced by paramagnetic Mn^2+^ impurities [16,17] acting as probes for detection of the tunneling process. For DMAZn:Mn, the main tunneling signals were observed close to 2 MHz and 0.3 MHz, while for DMACd:Mn they appeared at about 0.7 MHz [17]. This allowed us to distinguish small differences in the rotational barriers of the DMA methyl groups in both systems, demonstrating the sensitivity of this method. In addition, we observed splittings of the spectral lines suggesting different methyl group environments were likely due to crystal defects and the soft nature of the hybrid perovskites.

In general, paramagnetic Mn^2+^ centers are well-suited replacements of divalent diamagnetic ions for detection of tunneling ESEEM due to their relatively slow spin-lattice relaxation and decoherence. In addition, in contrast to some other transition metals such as Cu^2+^ [19], these ions are not susceptible to Jahn–Teller distortion, which may significantly affect the rotational potential of neighboring methyl groups. On the other hand, tunneling ESEEM may provide information on such local lattice distortions created by various impurities in hybrid perovskites [20,21,22,23] and other materials, which is essential for understanding resilience and stability of different frameworks and the role of the defects on the dynamics and ordering of the molecular cations. This stimulates further studies on the detection of the methyl group tunneling using alternative paramagnetic centers, which exhibit different structural and relaxation properties.

Here, we explore the feasibility of fast-relaxing Co^2+^ ions to study the methyl group tunneling using DMAZn hybrid perovskite as an exemplary system. DMAZn consists of the ZnO_6_ octahedra joined by the formate linkers into cuboid cavities containing DMA cations (see Figure 1b), which become ordered in the low-temperature phase (phase transition at about 160 K) [7,19,24,25,26,27]. We use X-, Q- and W-band EPR spectroscopy to show that Co^2+^ ions replace Zn^2+^ in DMAZn forming a high-spin state, which exhibits rapid relaxation and decoherence. We obtain the tunneling frequency and the rotational barrier of the methyl groups in DMAZn:Co by simulating the experimental ESEEM spectra using the density operator simulations. The obtained results are compared with the previously reported Mn^2+^ case revealing sensitivity of this method to resolve minute differences in the range of a couple % of the methyl group rotational barriers perturbed by different paramagnetic centers.

## 2. Results and Discussion

### 2.1. Spin Hamiltonian

**Figure 2:**
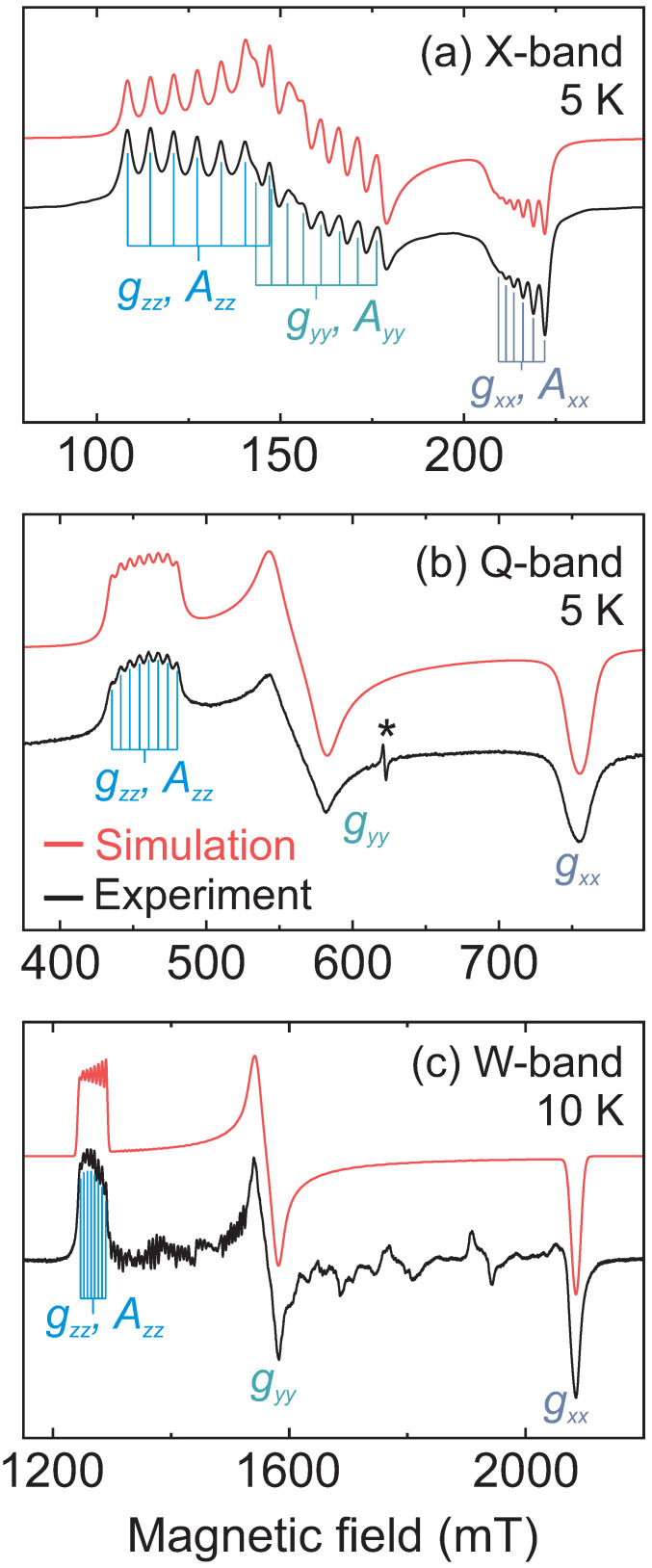
Measured (black) and simulated (red) CW EPR spectrum of DMAZn:Co obtained at (**a**) X- (5 K), (b) Q- (5 K) and (**c**) W-band (10 K). Asterisk in (**b**) marks unassigned impurity signal at g∼3.90. Signals unaccounted by the simulation in (**c**) originate from a non-ideal powder average caused by a limited number of crystallites.

First, we used CW EPR spectroscopy to characterize the Co^2+^ centers in DMAZn hybrid perovskite. The measured X-, Q- and W-band CW EPR spectra of DMAZn:Co powder obtained at low temperature are presented in Figure 2, revealing a typical EPR powder pattern of a high-spin Co^2+^ (electron configuration 3d${}^{7}$) having a rhombic g tensor [28,29,30,31,32]. The high-spin state of Co^2+^ is in agreement with the observations in pure DMACo hybrid perovskite [33]. The observed EPR lines are further split by the hyperfine interaction between the unpaired electrons and nucleus of the ^59^Co isotope (100% natural abundance). Note that we also observed traces of unintentionally incorporated Mn^2+^ impurities (see Figure S2), which were analyzed in detail in previous studies where they had been introduced intentionally [16,21,25,26,34,35].

The high-spin Co^2+^ center has three unpaired electrons, and hence the total electron spin is S=3/2. In addition, the orbital angular momentum of this ion is not quenched (L=3) leading to a strong spin-orbit coupling [28,30,36,37]. This interaction causes a very high zero-field splitting between the two lowest Kramers doublets, and thus the paramagnetic center can be effectively described using the effective electron spin-1/2 formalism [28,32,37].

We simulated the observed Co^2+^ spectra of DMAZn:Co using the effective spin Hamiltonian [28,32,37]:

(1) $$H_{\mathrm{eff}}=\beta_{e}{\mathrm{Bg}}_{\mathrm{eff}}S+{\mathrm{SA}}_{\mathrm{eff}}I,$$

where the first term describes the electron Zeeman interaction characterized by the effective electron spin S=1/2 and the effective $g_{\mathrm{eff}}$ tensor of the Co^2+^ center. The second term takes into account the hyperfine interaction between the electron spin and the nuclear spin I=7/2 of ^59^Co. This interaction is described by the effective hyperfine tensor $A_{\mathrm{eff}}$. B and $\beta_{e}$ denote the external magnetic field and Bohr magneton, respectively.

The simulated spectra, presented in Figure 2, reveal a very good agreement with the experiments for all three frequency bands. Note that the same set of the spin Hamiltonian parameters was used for the three simulations. The determined principal components of $g_{\mathrm{eff}}$ are $g_{\mathrm{eff},xx}=3.223\left(9\right)$, $g_{\mathrm{eff},yy}=4.312\left(3\right)$, and $g_{\mathrm{eff},zz}=5.304\left(2\right)$ with a mean value of 4.28(1), which is typical for high-spin Co^2+^ [29,30,31,36,38], supporting our assignment of the spin state. The effective ^59^Co hyperfine tensor obtained from the simulations has the following principal components: $A_{\mathrm{eff},xx}=95\left(2\right)$ MHz, $A_{\mathrm{eff},yy}=284\left(5\right)$ MHz and $A_{\mathrm{eff},zz}=480\left(1\right)$ MHz, which also fall within the expected range of values for high-spin Co^2+^ in an octahedral environment [30,31].

The effective components can be used to calculate the real g and A tensors of the full spin Hamiltonian (S=3/2) [28,32,37,38] yielding g=[2.156(2),2.652(1),3.223(9)] and A=[142(3),240(1),95(2)] MHz. The rhombic g tensor indicates that Co^2+^ centers in DMAZn occupy a low-symmetry environment, which is in agreement with the low-temperature structures of both DMAZn and DMACo which show distorted metal-oxygen octahedra [7,39]. The spectral simulations obtained using the S=3/2 model with real g and A tensors reproduce the results of the spin-1/2 approach for a very high value of the splitting Δ between the two lowest Kramers doublets (Δ≳500 GHz). A very high value of Δ is in agreement with our simulations of the W-band CW EPR spectrum, where we did not observe any deviations from the effective spin-1/2 model, indicating Δ≫95 GHz.

To assess the value of Δ, we performed temperature-dependent X-band CW EPR experiments of DMAZn:Co (see Figure S3). We observed that with increasing temperature, the signal intensity starts to decrease, and the spectrum completely disappears at about 75 K indicating fast relaxation of the Co^2+^ centers. The temperature at which the signal vanishes can be used to estimate Δ[30] providing Δ∼1.5 THz, which is much higher than our measurement frequencies.

We also recorded the spectrum of DMAZn:Co using pulsed EPR at different frequency bands and low temperature. The obtained EDFS spectra (see Figure S4) show both Co^2+^ and Mn^2+^ impurities, allowing us to perform further characterization using more advanced pulsed EPR experiments.

### 2.2. Relaxation Properties

**Figure 3:**
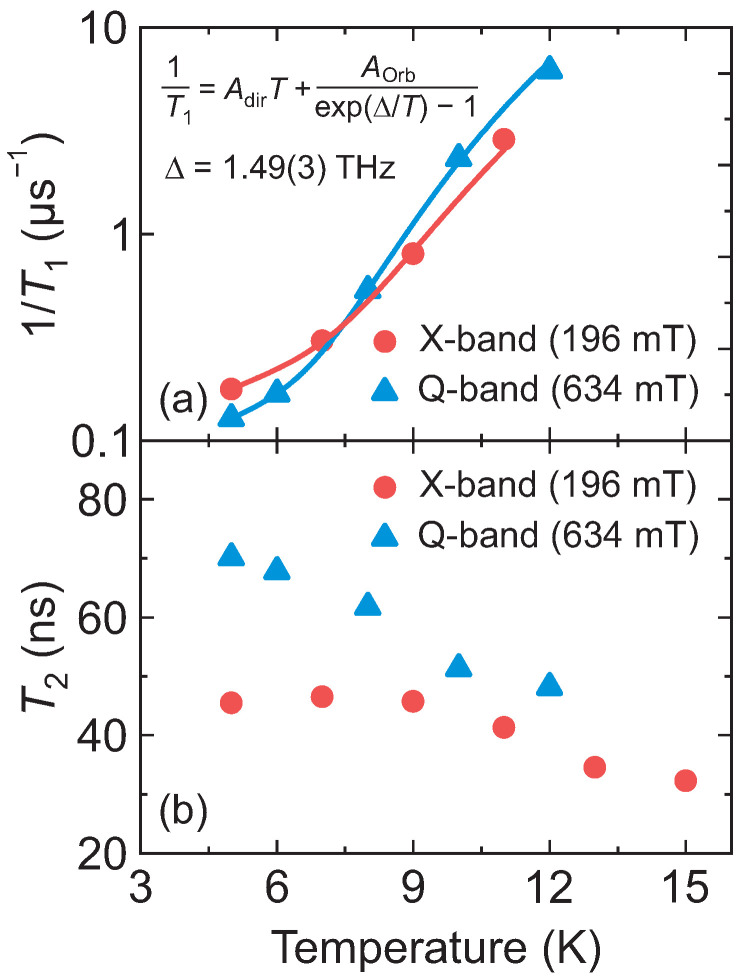
Temperature dependence of the (**a**) spin-lattice relaxation rate $1/T_{1}$ and (**b**) electron spin coherence time $T_{2}$ of DMAZn:Co obtained at X- (196 mT) and Q-band (634 mT) frequencies. Error bars are smaller than data points. Solid curves in (**a**) represent the best fit to the combination of the direct and Orbach relaxation model.

We used pulsed EPR to study the relaxation properties of the Co^2+^ centers in DMAZn:Co, which are important for a subsequent investigation of the methyl group tunneling. The spin-lattice relaxation time $T_{1}$ was obtained by fitting a stretched exponential function to the experimental data using the same stretching factor γ=0.41 for both frequency bands (see Figure S5), indicating a nearly-Gaussian distribution [40] of $T_{1}$ in DMAZn:Co. Note that a similar type of distribution was also observed in other hybrid perovskites [16,41]. The obtained temperature dependence of the relaxation rate $1/T_{1}$ is presented in Figure 3a, exhibiting a fast increase with increasing temperature in the range from 5 K to 12 K. Such a rapid relaxation is typical for high-spin Co^2+^ and is mainly governed by the Orbach (two phonon) process [42,43]. This process involves a high-lying excited spin state, and thus it can be used to determine the splitting Δ between the two lowest Kramers doublets.

We approximated the obtained temperature dependence of the relaxation rate $1/T_{1}$ using a combination of the Orbach and direct (single phonon) processes [36]:

(2) $$1/T_{1}=A_{\mathrm{dir}}T+\frac{A_{\mathrm{Orb}}}{\exp(\Delta /T)-1}.$$

Here, $A_{\mathrm{dir}}$ and $A_{\mathrm{Orb}}$ correspond to the contributions of both processes. A global fit, with respect to the splitting Δ, was performed for both frequency data sets revealing a perfect agreement with the experimental data (see Figure 3a). The obtained value of Δ of 1.49(3) THz perfectly agrees with the value determined from the temperature-dependent CW EPR experiments (Figure S3), further supporting our interpretation. Other fit parameters are $A_{\mathrm{dir}}=3.54\left(2\right)\times 10^{-5}\;K^{-1}{\mathrm{ns}}^{-1}$, $A_{\mathrm{Orb}}=1.4\left(2\right)\;{\mathrm{ns}}^{-1}$ (X-band), and $A_{\mathrm{dir}}=2.53\left(1\right)\times 10^{-5}\;K^{-1}{\mathrm{ns}}^{-1}$, $A_{\mathrm{Orb}}=2.5\left(5\right)\;{\mathrm{ns}}^{-1}$ (Q-band).

The temperature dependence of the electron spin coherence time $T_{2}$ of DMAZn:Co obtained at X- and Q-band frequencies is presented in Figure 3b (see Figure S6 for the Hahn echo decays). The value of $T_{2}$ measured at X-band decreases from about 45 ns at 5 K to about 30 ns at 15 K, which is the detection limit of our spectrometer, while the Q-band $T_{2}$ is slightly longer in the whole temperature range. Note that the $T_{2}$ times at both frequency bands are significantly shorter than $T_{1}$ indicating a different cause of the decoherence such as the zero-field splitting. We also performed measurements of the instantaneous diffusion suppression [44], which revealed no changes in the $T_{2}$ time indicating that it is not limited by the interactions between the Co^2+^ ions.

We note that the observed $T_{1}$ and $T_{2}$ values of Co^2+^ in DMAZn are significantly shorter compared with the slow-relaxing transition metal ions such as Mn^2+^ (e.g., $T_{1}∼100$ μs and $T_{2}∼1$ μs in DMAZn:Mn at 10 K [16]). Such short relaxation times may pose problems for detection of the methyl group tunneling using ESEEM, which is tested in the following.

### 2.3. Methyl Group Tunneling

**Figure 4:**
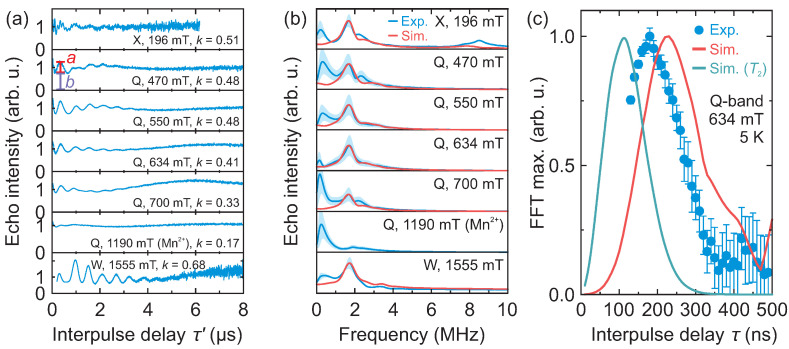
(**a**) Background corrected 3p ESEEM time-domain traces of DMAZn:Co obtained at 5 K and at different microwave frequencies and magnetic fields. The interpulse delay τ was 130 ns (X- and Q-band) and 200 ns (W-band). Parameters *a* and *b* indicated on the second trace are used to define the modulation depth as k=a/(a+b). (**b**) Corresponding experimental and simulated frequency-domain spectra. The shaded regions mark the uncertainty obtained using different levels of apodization. (**c**) τ-dependence of the frequency-domain signal obtained at 643 mT (Q-band) and 5 K. Solid curves show simulation obtained using $\nu_{t}=1.84$ MHz and the effective g=3.828, with and without the $T_{2}$ relaxation taken into account.

We performed X-, Q- and W-band 3p ESEEM experiments of DMAZn:Co to investigate the feasibility of Co^2+^ centers to detect the methyl group tunneling. The obtained 3p ESEEM time-domain traces are presented in Figure 4a (also see Figure S7) revealing pronounced ESEEM oscillations. The corresponding frequency-domain spectra (Figure 4b) show a peak at 1.71 MHz, which is not affected by the magnetic field. Such a field independent ESEEM is a distinct signature of the methyl group tunneling, as revealed in our previous study [17]. This is further supported by the 3p ESEEM experiments performed at different τ values showing a buildup of the ESEEM modulation [17], as revealed in the amplitude of the frequency-domain spectra (Figure 4c).

In addition to the tunneling signals around 1.7 MHz, we also detected a low-frequency line in the frequency range of 0.15–0.3 MHz (see Figure 4b). The amplitude of this signal is sensitive to the background correction and apodization level suggesting that it may be an artefact due to non-ideal data processing. To investigate this further, we performed analysis of the time-domain data with varying apodization to obtain the confidence region of the frequency-domain signals (Figure 4b). Our results show that this signal can be partially suppressed, but not fully removed using different background functions. An alternative explanation for this low-frequency line may be the transition between the hyperfine-perturbed $E^{\prime }$ and $E^{\prime \prime }$ states (see Figure 1a), although its intensity is expected to be much lower [17]. Note that similar 3p ESEEM signals in the low-frequency part of the spectrum were also observed for the DMAZn:Mn case [17].

We used density operator calculations to simulate the tunneling ESEEM of DMAZn:Co. In the simulations, we assumed the effective spin-1/2 model, while the hyperfine couplings between the Co^2+^ center and protons of the 16 nearest methyl groups were calculated using the recently reported low-temperature structure of DMAZn [7] (see Simulation details). The tunnel frequency $\nu_{t}$ was the only free parameter in the simulations. The simulated ESEEM spectra (see Figure S8 for the time-domain data) obtained using $\nu_{t}=1.84\left(2\right)$ MHz are presented in Figure 4b, revealing a very good agreement with the experimental data for all measured field and frequency values. Figure S9 shows the individual time-domain contribution for each of the 16 methyl groups used in the simulations, indicating that only the eight nearest groups have a noticeable effect, while the dominant contribution arises from the two closest groups that point to the Co^2+^ ion.

We also investigated how well our simulations reproduce the modulation depth parameter k=a/(a+b) (see Figure 4a for the definition of *a* and *b*) of the time-domain signals. Depending on the frequency and magnetic field, the measured value of *k* ranges from 0.33 to 0.68, with a mean value of 0.48 (Figure 4a). The simulated modulation depth varies from 0.42 to 0.86 (mean 0.64) (see Figure S8), which is in satisfactory agreement with the experiment. In both cases, the highest modulation depth is observed at W-band frequency. Slightly lower measured values point to a distribution of the tunneling frequency caused by different methyl group environments, which may originate from the structural defects [45] and local deformations due to the soft nature of the hybrid perovskite [46].

Our simulations rather well reproduced the experimentally observed buildup of the modulation (Figure 4c). By taking into account the $T_{2}$ relaxation, which occurs when the interpulse delay τ is incremented, the simulated FFT maximum appears at about 120 ns, which is 60 ns lower compared with the experiment. This discrepancy may indicate that a full spin Hamiltonian approach is needed to fully reproduce all aspects of the experimental results. However, determination of this Hamiltonian requires either EPR experiments at very high frequency [32] or sophisticated calculations, both of which are beyond the scope of this work. Note that the ESEEM spectra simulated using the effective and real *g*-factors are very similar (see Figure S10), indicating that the spin-1/2 formalism is sufficient to extract the tunnel frequency $\nu_{t}$.

The tunnel frequency can be used to calculate the rotational barrier $V_{3}$ of the methyl groups [2]. The obtained value of 1.84(2) MHz for DMAZn:Co translates to $V_{3}=10.60\left(2\right)$ kJ/mol, which is in a reasonable agreement with the density functional theory (DFT) prediction of 12.3 kJ/mol reported in our previous work [17]. We aimed to compare this result with the rotational barrier determined using the unintentionally doped traces of Mn^2+^ in DMAZn:Co. However, we observed that the tunneling ESEEM signal probed by Mn^2+^ impurities in DMAZn:Co is significantly broader compared to our previous study [17], where Mn^2+^ ions were intentionally doped into the structure of DMAZn (see Figure S11 for comparison). We assume that this additional broadening occurs due to faster relaxation of Mn^2+^ centers induced by Co^2+^ ions that are simultaneously present within the crystal structure.

**Figure 5:**
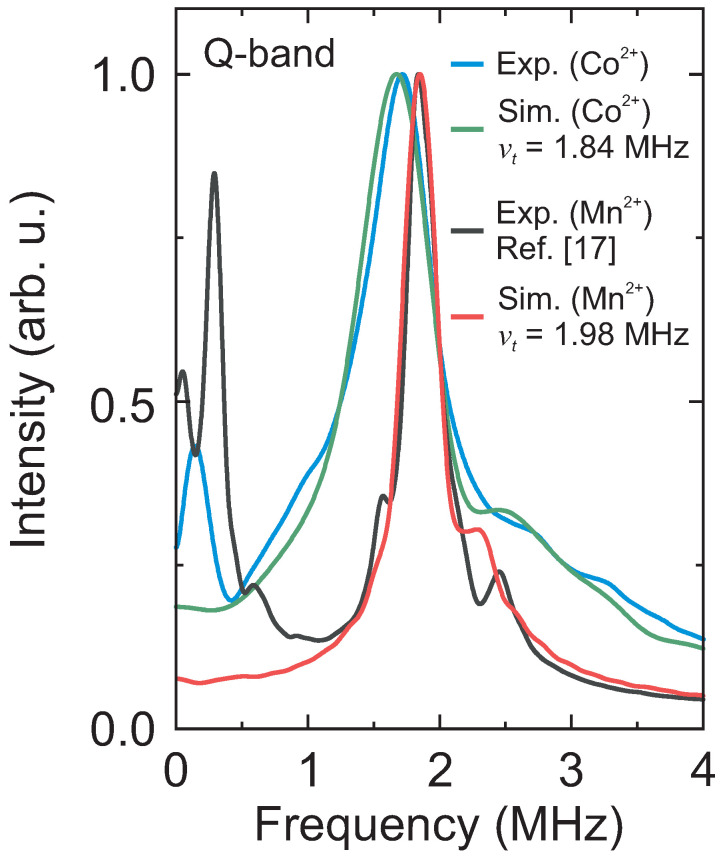
Comparison of the Q-band ESEEM spectra of DMAZn:Co (634 mT, 5 K) and DMAZn:Mn (1194 mT, 10 K, see also Ref. [17]) and their corresponding simulations. The same data analysis routine was employed in both cases. Simulation parameters: $\nu_{t}=1.84$ MHz, g=3.828 (DMAZn:Co), and $\nu_{t}=1.98$ MHz, g=2.0005 [26] (DMAZn:Mn).

Thus, for a better comparison of both paramagnetic centers, we decided to use the ESEEM data of the previously reported DMAZn:Mn system [17]. The experimental ESEEM spectra of DMAZn:Co and DMAZn:Mn are presented in Figure 5 revealing that the main tunneling signal obtained using the Mn^2+^ centers occurs at about 120 kHz higher frequency. We also used the same procedure as for the DMAZn:Co case to simulate the Mn^2+^ spectrum, which provided a tunnel frequency of $\nu_{t}=1.98\left(1\right)$ MHz. Note that this value is slightly higher than reported in our previous work ($\nu_{t}=1.93$ MHz) [17], where the simulations were performed using a less sophisticated structural model based on the crystal structure of the related DMAMn hybrid perovskite.

The obtained $\nu_{t}=1.98\left(1\right)$ MHz translates to the methyl group rotational barrier of 10.50(1) kJ/mol, which is only slightly lower compared to the Co^2+^ case (10.60(1) kJ/mol). Such a difference may originate from a higher degree of lattice distortion caused by the high-spin Co^2+^ ions, which, in contrast to the high-spin Mn^2+^, can exhibit a pseudo Jahn–Teller effect. The ability to reliably resolve minute changes (of the order of 1%) in $V_{3}$ introduced by different paramagnetic centers demonstrates an incredible sensitivity of the tunneling ESEEM recorded with the 3p ESEEM pulse sequence to probe the local methyl group environment.

## 3. Experimental and Simulation Details

### 3.1. Sample Synthesis and Characterization

ZnCl_2_ (99.999%, Sigma-Aldrich, St. Louis, MO, USA), CoCl_2_·xH_2_O (99.999%, Sigma-Aldrich), a 2.0 M solution of (CH_3_)_2_NH in methanol (Sigma-Aldrich), methanol (99.8%, Sigma-Aldrich) and formic acid (98%, Fluka, Switzerland) were commercially available and used without further purification. Crystals of DMAZn: 1 Co^2+^ mol% were grown by a slow diffusion method. In a typical experiment, 2.5 mL of 2.0 M solution of (CH_3_)_2_NH in methanol and 0.5 mL of formic acid were added to 10 mL of methanol. This solution was placed at the bottom of a glass tube (20 mm inner diameter). To this solution, 2 mL of methanol was layered, followed by 20 mL of methanol solution containing 0.99 mmol of ZnCl_2_ and 0.01 mmol of CoCl_2_·xH_2_O. The tube was sealed and kept undisturbed. The pink crystals were harvested after 4 days, washed three times with methanol, and dried at room temperature.

The preservation of the perovskite phase after doping with Co was confirmed by the Raman spectroscopy (see Figure S1) [47]. Raman spectra were measured using a Bruker FT 100/S spectrometer with Nd:YAG laser excitation (1064 nm). The spectral resolution was 2 cm${}^{-1}$.

### 3.2. EPR Spectroscopy

The synthesized DMAZn:Co crystals were ground into a fine powder, which was placed into 4, 1.6 and 0.9 mm outer diameter EPR tubes for measurements at X- (9.5 GHz), Q- (34 GHz) and W-band (94 GHz) frequencies, respectively. For X- and Q-band EPR experiments, we used a Bruker ELEXSYS E580/IF-Q EPR spectrometer equipped with Bruker ER4118X-MD5 (pulsed X-band), high-Q ER4102ST (CW X-band) and EN5107D2 (pulsed/CW Q-band) microwave resonators. High-power pulses were obtained using 1 kW TWT (X-band) and 10 W solid-state (Q-band) microwave amplifiers. The EPR experiments at W-band were carried out using a Bruker ELEXSYS E680 X-/W-band spectrometer equipped with an EN 680-1021H resonator and a 2 W solid-state amplifier. The helium flow cryostats were used to stabilize the temperature.

For the CW EPR experiments, we used the following amplitude and frequency of the modulation field: 100 kHz and 1 G (X-band), 50 kHz and 1 G (Q-band), and 100 kHz and 4 G (W-band). The microwave power was adjusted to avoid saturation of the EPR signal.

The pulsed EPR experiments of DMAZn:Co were performed using 16 ns π/2- and 32 ns π-pulses. The echo-detected field sweep (EDFS) spectra were recorded using a Hahn echo pulse sequence (π/2−τ−π−τ−echo) with an interpulse delay τ of 130 ns (X- and Q-band) and 200 ns (W-band). The three-pulse (3p) ESEEM was recorded by integrating the stimulated echo obtained using the $\pi /2-\tau -\pi /2-\tau^{\prime }-\pi /2-\tau -\mathrm{echo}$ pulse sequence, where the interpulse delay $\tau^{\prime }$ was incremented by a time step of 6 ns (X- and Q-band) and 4 ns (W-band). The spin-lattice relaxation time $T_{1}$ was obtained using the inversion recovery pulse sequence ($\pi -\tau^{\prime }-\pi /2-\tau -\pi -\tau -\mathrm{echo}$), while the spin decoherence time $T_{2}$ was measured using the Hahn echo experiment. Four-step phase cycling was used to cancel unwanted echoes except for the two-pulse experiments, where two-step phase cycling was employed.

For the data analysis, the experimental 3p ESEEM time-domain traces were divided by bi-exponential decay functions followed by Hamming apodization, zero-filling and fast Fourier transform with cross-term averaging [48] to yield the frequency-domain spectra. The values of $T_{1}$ relaxation time were obtained by fitting a stretched exponential function ($V=a(1-b\exp{\left(-\tau^{\prime }/T_{1}\right)}^{\gamma })$) to the inversion recovery data, which provided a significantly better agreement compared to the single and bi-exponential recovery functions. The $T_{2}$ times were extracted from the Hahn echo decay experiments using a single exponential decay ($V=a\exp(-2\tau /T_{2})$). All steps were carried out using home-written MATLAB R2021b (The MathWorks Inc., Natick, MA, USA) scripts.

### 3.3. Simulation Details

We used EasySpin 5.2.33 [49] running on MATLAB R2021b (The MathWorks Inc.) to simulate the CW EPR spectra.

The 3p ESEEM signals of the methyl group tunneling were simulated using density operator formalism in home-written MATLAB scripts [17,50]. Spin-operator matrices and orientation grids were generated using EasySpin *sop* and *sphgrid* functions. The hyperfine interactions between the Co^2+^ center and methyl protons were calculated using the point-dipole approximation from the low-temperature structure of DMAZn [7]. The eight nearest DMA cations (16 methyl groups) were considered during the simulations. For each orientation, a product of the simulated time-domain traces of all 16 groups was obtained as for ordinary 3p ESEEM. The resulting time-domain signal was obtained by calculating a weighted average of the traces obtained at different orientations. The baseline was removed by normalizing the signal to its average value.

The rotational barrier $V_{3}$ was calculated from the tunnel frequency $\nu_{t}$ by diagonalizing the rotational Hamiltonian in a basis of the free quantum rotor [2]. The tunnel frequency was taken as the energy difference between the two lowest-energy states.

## 4. Summary and Conclusions

In this work, we used EPR spectroscopy to study the methyl group tunneling in DMAZn hybrid perovskite doped with a small amount of paramagnetic Co^2+^ impurities. Our CW EPR experiments revealed that Co^2+^ centers occupy a low-symmetry site in the DMAZn structure forming a high-spin state, the lowest lying doublet of which can be well described using the effective spin-1/2 formalism. We observed a fast decrease of $T_{1}$ with increasing temperature, which was explained by the Orbach relaxation process with a 1.5 THz splitting between the two lowest Kramers doublets. The obtained $T_{2}$ time of DMAZn:Co also proved to be very short, of the order of tens of nanoseconds.

We used 3p ESEEM spectroscopy to study the feasibility of these fast-relaxing Co^2+^ ions to facilitate detection of the methyl group tunneling in DMAZn. A pronounced field-independent tunneling signal was observed at about 1.7 MHz, which we reproduced using simulations based on density operator formalism with a tunnel frequency of 1.84(2) MHz. This value translates to a rotational barrier $V_{3}$ of 10.60(2) kJ/mol. A comparison with the DMAZn:Mn case allowed us to resolve very small differences (in the range of a couple %) of the methyl group rotational barriers in the presence of Co^2+^ and Mn^2+^ paramagnetic centers in DMAZn.

In general, our results show that ESEEM of Co^2+^ centers can be successfully employed to study tunneling of the neighboring methyl groups in hybrid perovskites and other materials. In addition, we demonstrated that this spectroscopic approach is highly effective in determining minute differences in the rotational potentials of the methyl groups, providing a sensitive handle to probe their local environment. In terms of hybrid perovskites, this method could be used to investigate small structural distortions caused by different framework impurities, which otherwise are difficult to study.

## Data Availability

The data presented in this study are available on request from the corresponding author.

## Supplementary Materials

The following supporting information can be downloaded at: https://www.mdpi.com/article/10.3390/molecules28030979/s1, Figure S1: Raman spectra of DMAZn:Co and DMAZn, Figure S2: Additional Q-band CW EPR spectrum, Figure S3: Additional X-band CW EPR spectra, Figure S4: EDFS spectra of DMAZn:Co, Figure S5: Inversion recovery data of DMAZn:Co, Figure S6: Hahn echo decays of DMAZn:Co, Figure S7: Experimental 3p ESEEM time-domain traces of DMAZn:Co, Figure S8: Simulated 3p ESEEM time-domain traces of DMAZn:Co, Figure S9: Simulated 3p ESEEM time-domain traces of the nearest 16 methyl groups, Figure S10: Additional simulations of the frequency-domain spectrum, Figure S11: Additional 3p ESEEM spectrum.

## Abbreviations

The following abbreviations are used in this manuscript:

DMAZn	Dimethylammonium zinc formate
DMACo	Dimethylammonium cobalt formate
DMAMn	Dimethylammonium manganese formate
3p ESEEM	Three pulse electron spin echo envelope modulation
EPR	Electron paramagnetic resonance
CW	Continuous wave
ENDOR	Electron nuclear double resonance
NMR	Nuclear magnetic resonance
DFT	Density functional theory
