# Supplementary material for: Probing Methyl Group Tunneling in [(CH3)2NH2][Zn(HCOO)3] Hybrid Perovskite Using Co2+ EPR
Source: Molecules. 2023 Jan 18;28(3):979. doi: 10.3390/molecules28030979 (PMC9920925; doi:10.3390/molecules28030979)
Supplement: Supplementary file 1 [file molecules-28-00979-s001.zip › molecules-2146505-supplementary.pdf]

Supplementary Materials

Probing methyl group tunneling in $[(CH_3)_2NH_2][Zn(HCOO)_3]$ hybrid perovskite using Co^{2+} EPR

Gediminas Usevičius, Andrea Eggeling, Ignas Pocius, Vidmantas Kalendra, Daniel Klose, Mirosław Mączka, Andreas Pöppl, Jūras Banys, Gunnar Jeschke, and Mantas Šimėnas*

E-mail: mantas.simenas@ff.vu.lt

Additional Raman Data

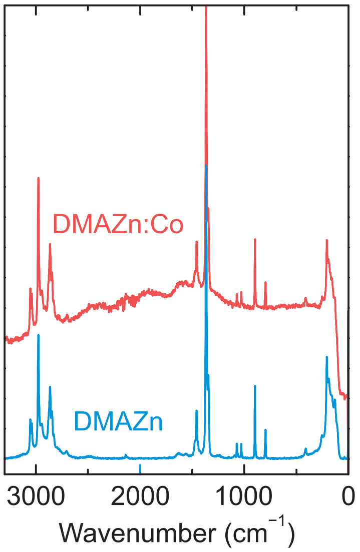

Figure S1: Raman spectra of undoped DMAZn and Co-doped DMAZn:Co obtained at room temperature.

Additional EPR Data

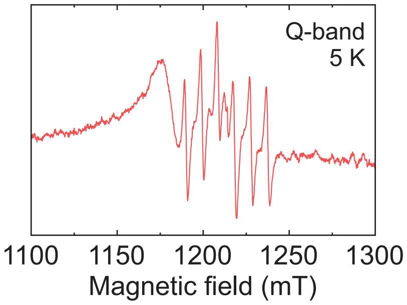

Figure S2: Q-band CW EPR spectrum of Mn^{2+} impurities in DMAZn:Co obtained at 5 K.

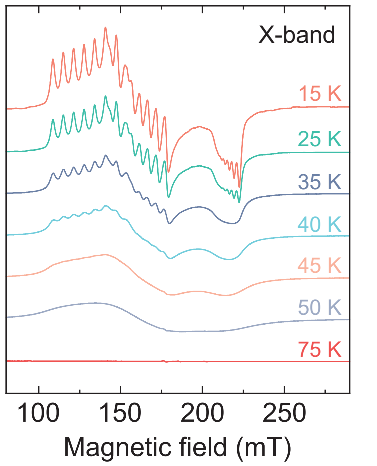

Figure S3: Temperature dependence of the X-band CW EPR spectrum of DMAZn:Co.

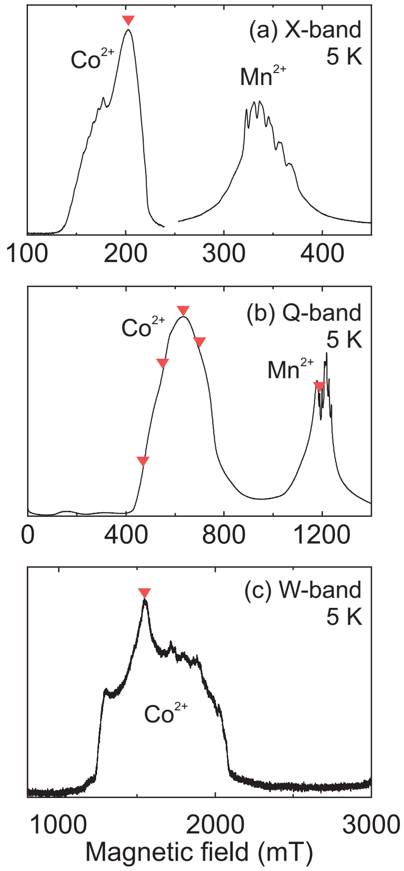

Figure S4: EDFS spectrum of DMAZn:Co obtained at 5 K and (a) X-, (b) Q-, and (c) W-band. Red triangles mark field positions, at which the 3p ESEEM experiments were performed. In addition to Co^{2+}, the X- and Q-band spectra also indicate the Mn^{2+} impurities.

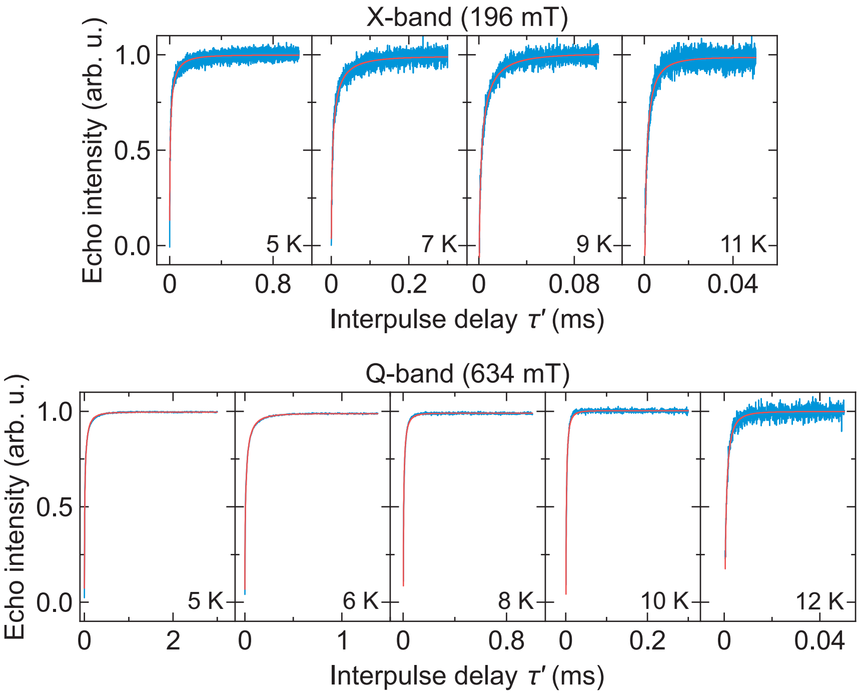

Figure S5: Inversion recovery of DMAZn:Co obtained at X- (196 mT) and Q-band (634 mT) frequencies and different temperatures. Red curves indicate the best fits to a stretched exponential recovery function: $V = a(1 - b\exp(-\tau'/T_1)^{\gamma})$, where $\gamma = 0.405(4)$ was obtained from a global fit of all curves.

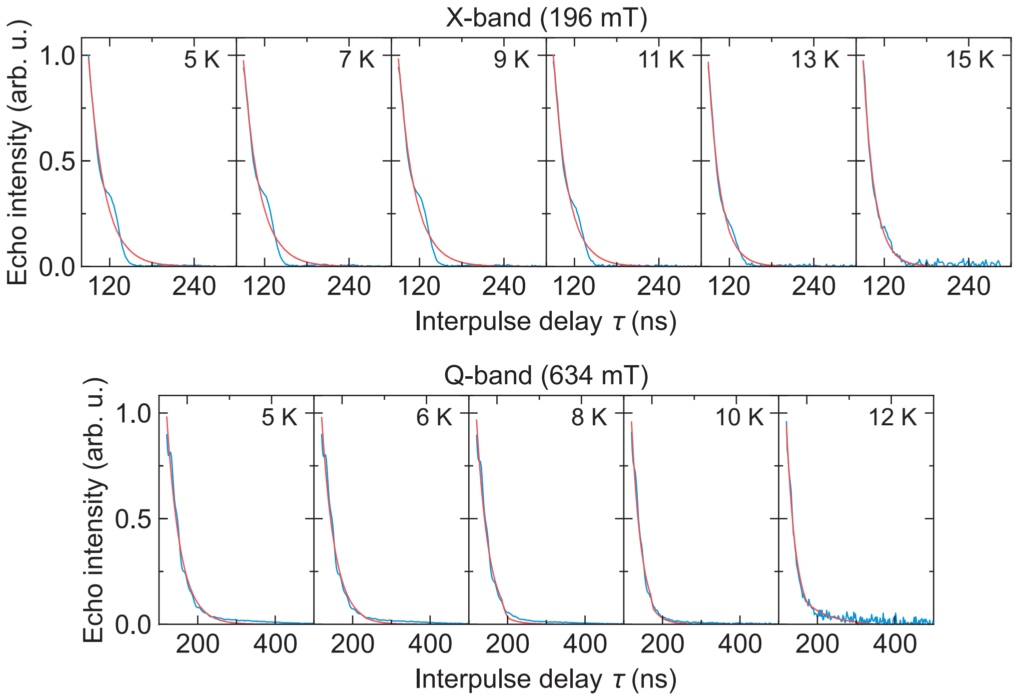

Figure S6: Hahn echo decay of DMAZn:Co obtained at X- (196 mT) and Q-band (634 mT) frequencies and different temperatures. Red curves indicate the best fits to a single exponential decay function.

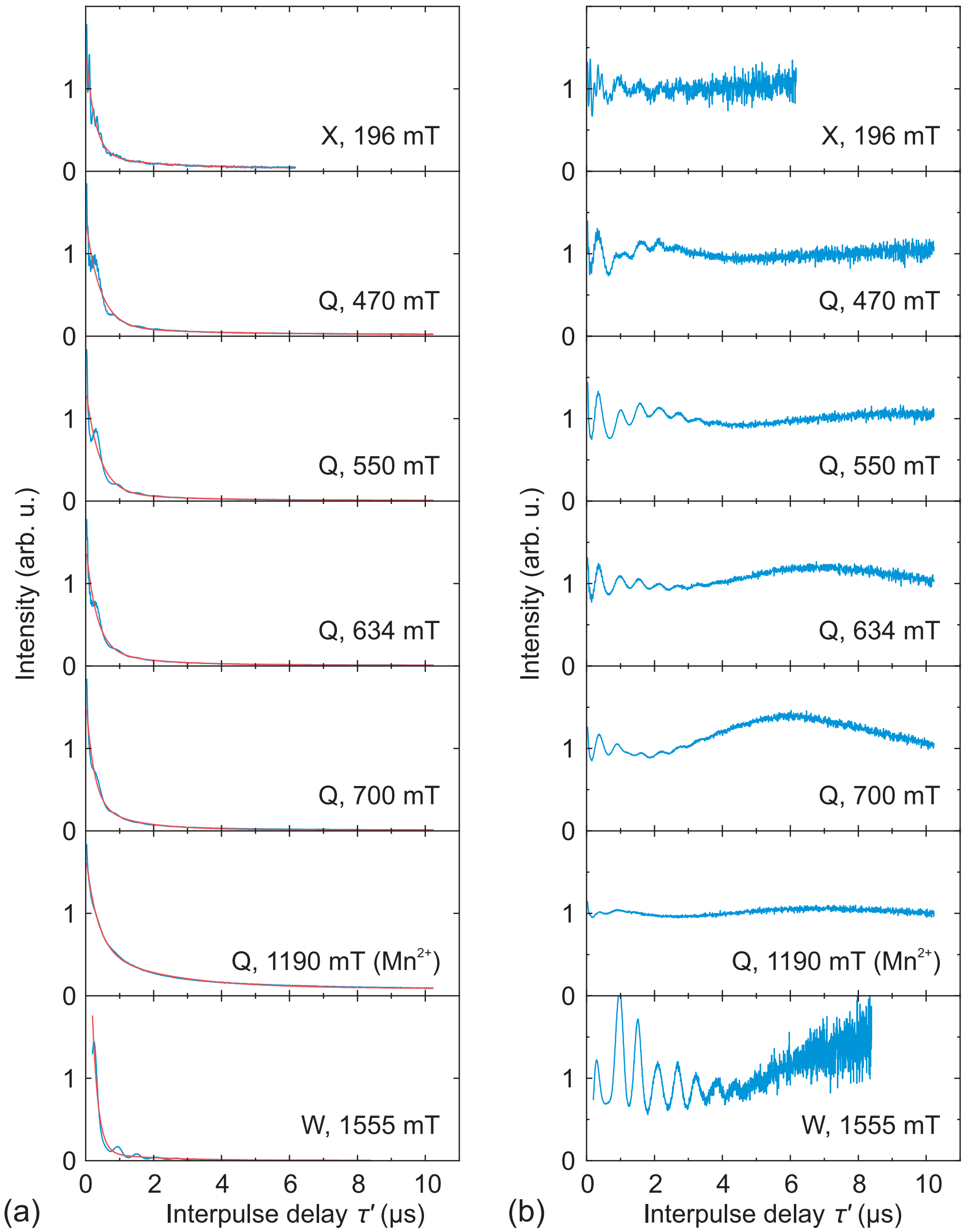

Figure S7: (a) Experimental 3p ESEEM time-domain traces of DMAZn:Co recorded at different frequency bands and magnetic fields. Red curves are the best fits to a bi-exponential decay function. (b) Time-domain traces obtained after division by the baseline function.

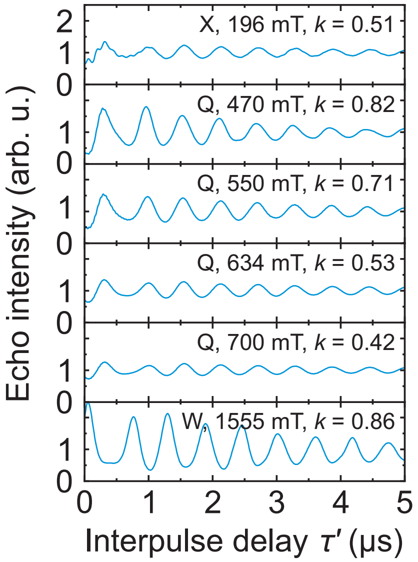

Figure S8: Simulated 3p ESEEM time-domain traces of DMAZn:Co at different microwave frequencies and magnetic fields using $\nu_t = 1.84$ MHz and the effective $g = 3.828$. The modulation depth parameters k were determined from the first oscillation.

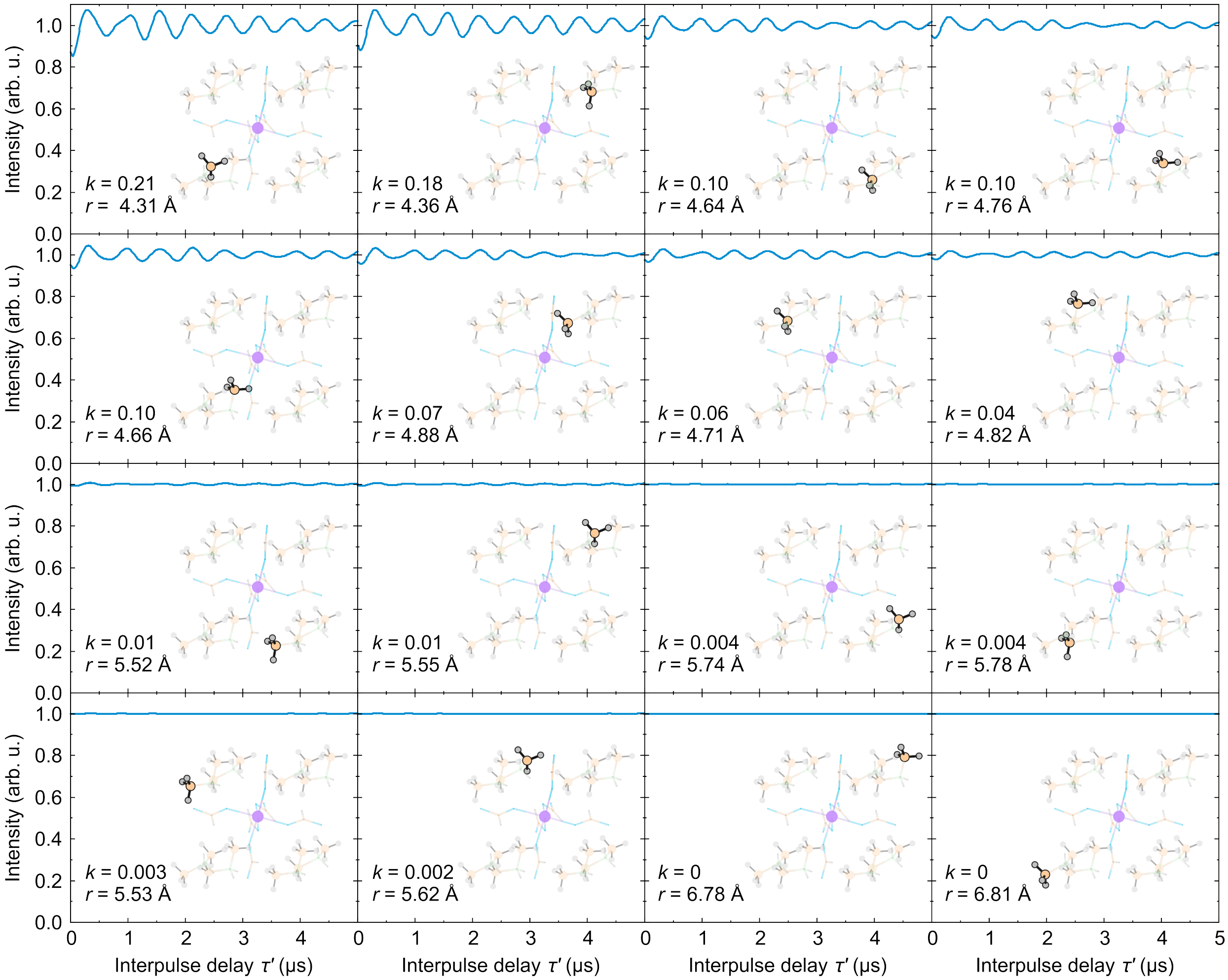

Figure S9: Simulated 3p ESEEM time-domain traces of the nearest 16 methyl groups surrounding a single Co^{2+} center in DMAZn:Co (see insets). Simulations performed at Q-band and 634 mT using $\nu_t = 1.84$ MHz and the effective $g = 3.828$. The modulation depth parameters k were determined from the first oscillation. The indicated distance is between the metal center and carbon atom of a methyl group.

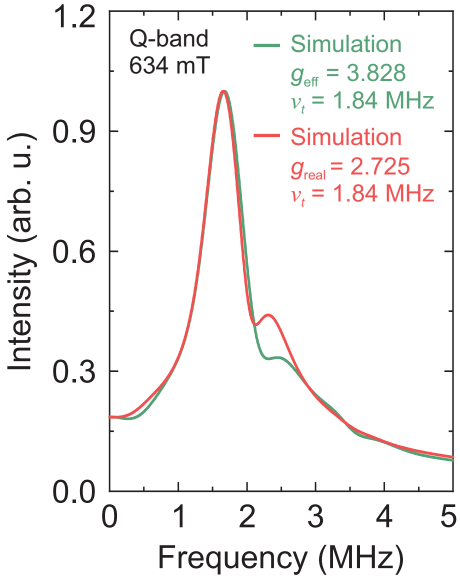

Figure S10: Simulated frequency-domain spectra of DMAZn:Co (643 mT, Q-band) obtained using the effective and real g-values of the Co^{2+} ions.

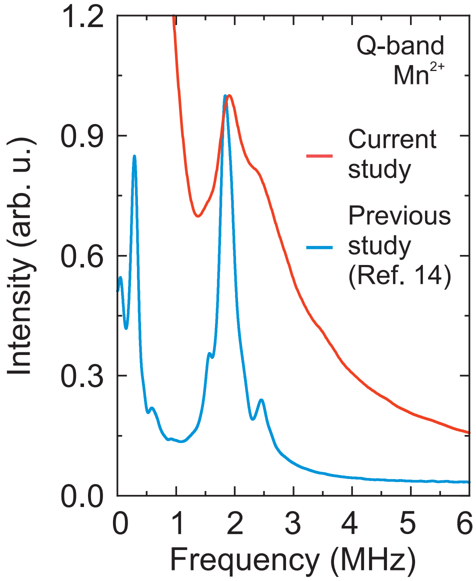

Figure S11: Comparison of the Q-band ESEEM spectrum of DMAZn obtained using the Mn^{2+} centers in our current and previous (Ref. 14) studies. A significant broadening of the ESEEM signal is observed in our current study, where we use unintentionally doped traces of Mn^{2+} to probe the methyl group tunneling.
